# ﻿Six new species of *Margattea* Shelford, 1911 (Blaberoidea, Pseudophyllodromiidae, Neoblattellini) from China

**DOI:** 10.3897/zookeys.1191.113147

**Published:** 2024-02-16

**Authors:** Qian-Qian Li, Wen-Wen Yao, Ke Zhang, Zong-Qing Wang, Yan-Li Che

**Affiliations:** 1 College of Plant Protection, Southwest University, Beibei, Chongqing 400715, China Southwest University Chongqing China; 2 Key Laboratory of Agricultural Biosafety and Green Production of Upper Yangtze River (Ministry of Education), Southwest University, Chongqing 400715, China Southwest University Chongqing China

**Keywords:** ABGD, DNA barcoding, *Margattea*, new species

## Abstract

Six *Margattea* species are established and described: three are cryptic species, namely, *M.parabisignata* Li & Che, **sp. nov.**, *M.semicircularis* Li & Che, **sp. nov.**, and *M.forcipata* Li & Che, **sp. nov.** They are distinguished from known species *M.bisignata*, *M.spinifera*, and *M.paratransversa* by their male genitalia with the aid of molecular species delimitation method (ABGD) using *COI* as the molecular marker. The other three new species are *M.pedata* Li & Che, **sp. nov.**, *M.undulata* Li & Che, **sp. nov.**, and *M.bisphaerica* Li & Che, **sp. nov.** Morphological and genitalia photographs of these new species of *Margattea*, as well as a key to the species of *Margattea* from China, are provided.

## ﻿Introduction

A total of 63 species of the genus *Margattea* in Neoblattellini have been recorded in Asia, Africa, and parts of Oceania (Beccaloni 2014), and 26 of them are known from China (He et al. 2021). There are twelve genera in Neoblattellini. *Margattea* is the only genus that is not distributed in the New World, while all the other eleven genera are only present in the New World. *Margattea* is morphologically similar to the genus *Balta* in the yellowish brown body, the subelliptical pronotum (sometimes with spots), and wings that usually exceed the end of the abdomen (He et al. 2021). Despite being a relatively well-diversified genus, only four molecular phylogenetic analyses sampled *Margattea* species (Evangelista et al. 2021, 2023; Liu et al. 2023; Wang et al. 2023). Due to the different molecular data and sampling taxa in molecular analyses listed above, the sister group of *Margattea* was inconsistently recovered, but *Balta* was always found phylogenetically distant from *Margattea*.

At present, members of *Margattea* are identified by the simple, cylindrical, and symmetrical styli, the usually specialized eighth abdominal tergum, the pronotal disc with symmetrical stripes and maculae, and the median phallomere usually with accessory structure (Roth 1989; Wang et al. 2009; Liu and Zhou 2011; He et al. 2021). However, these diagnostic characteristics can be found separately in other genera; moreover, the styli of *Margattea* are diverse: asymmetrical in *Margatteapseudolimbata* Wang, Li, Wang & Che, 2014 (Wang et al. 2014), symmetrical and foot-shaped styli or asymmetrical and spherical styli in other samples we examined. Therefore, it is still necessary to further determine the diagnostic characteristics of *Margattea*.

DNA barcoding (Hebert et al. 2003) has been widely used in the identification of cockroach species (Knebelsberger and Miller 2007; Evangelista et al. 2013; Wang et al. 2021), the estimation of cockroach species richness (Evangelista et al. 2014), the matching of individuals with sexual dimorphism (Yang et al. 2019; Deng et al. 2020; Han et al. 2022; Luo et al. 2023) and the discovery of cryptic species (Bai et al. 2018; Zhu et al. 2022). In this study, we combine molecular species delimitation methods with morphological characteristics, including male genitalia, to determine species found in China. In addition, the generic diagnosis of *Margattea*, mainly concerning the styli, left and median phallomere, is redefined after the examination of most *Margattea* species on the basis of the specimen or the original description (but not for *M.beauvoisii* (Walker, 1868), *M.baluensis* (Hanitsch, 1933), *M.bipunctata* (Hanitsch, 1933), *M.buitenzorgensis* Caudell, 1927, *M.centralis* (Gerstaecker, 1883), *M.crucifera* (Hanitsch, 1925), *M.diacantha* (Hebard, 1929), *M.gulliveri* Hanitsch, 1928, *M.importata* Bey-Bienko, 1964, *M.microptera* (Hanitsch, 1925), *M.nana* (Saussure, 1869), *M.nebulosa* (Shelford, 1907), *M.obtusifroms* (Walker, 1868), *M.philippinensis* (Roth, 1990), *M.remota* (Hebard, 1933), *M.sinclairi* Hanitsch, 1928, and *M.variegata* (Brunner von Wattenwyl, 1898)).

## ﻿Materials and methods

### ﻿Morphological study

All type specimens are deposited in College of Plant Protection, Southwest University, Chongqing, China (**SWU**). Male genital segments were immersed in 10% NaOH solution and incubated with water at 90 °C for 15 minutes to dissolve the fat. All segments were dissected and stored in glycerol for observation, and preserved along with the remainder of the specimen which is stored in ethyl alcohol. All photos were taken by a Leica DFC digital microscope camera attached to a Leica M205A stereomicroscope, then modified with Adobe Photoshop CC 2019. Specimens examined were measured by Vernier Caliper. Morphological terminology mainly follows Roth (2003). The sclerites of male genitalia mainly follows McKittrick (1964). The terminology of veins follows Li et al. (2018). Abbreviations of veins are as follows:

**ScP** subcosta posterior;

**R** radius;

**RA** radius anterior;

**RP** radius posterior;

**Pcu** postcubitus;

**M** media;

**CuA** cubitus anterior;

**CuP** cubitus posterior;

**V** vannal.

### ﻿DNA extraction, amplification, and sequencing

Total DNA was obtained from legs and thoracic muscle using the Hipure Tissue DNA Mini Kit, and the remainder of the specimen was stored in ethyl alcohol. The primers were used to amplify the 658 bp cytochrome c oxidase subunit I (*COI*) fragment (Table 1). The amplification conditions were: initial denaturation at 98 °C for 2 min, followed by 35 cycles for 10 sec at 98 °C, 10 s at 49–51 °C, and 3 min for 72 °C, with final extension of 3 min at 72 °C, then held at 4 °C. The amplified samples showing bands in agarose gels were sequenced by Beijing Tsingke Biotech Co., Ltd.

**Table 1.** Primers used in the molecular study.

Primers	Base sequence		Reference
	F	R	
*COI*-F6/R6	5’-CAACYAATCATAAAGANATTGGAAC-3’	5’-TAAACTTCAGGGTGACCAAARAATCA-3’	Yang et al. 2019
*COI*-F5/R5	5’-GGTCAACAAATCATAAGATATTGG-3’	5’-TAAACTTCAGGGTGACCAAAAAATCA-3’	Folmer et al. 1994

### Sequence processing and molecular analysis

A total of 105 *COI* sequences were analyzed, of which 53 sequences are obtained in this study and 52 sequences were downloaded from GenBank (*Baltavilis*, *Sorineuchoranigra*, and *Mantisreligiosa* were selected as outgroups) (Table 2). All sequences were aligned by MEGA 7 (Kumar et al. 2016) and adjusted after translation into amino acid sequences. The genetic divergence value was quantified based on Kimura 2-parameter (K2P) (Kimura 1980) by MEGA 7. The Maximum Likelihood (ML) tree was constructed in PhyloSuite v. 1.2.2 (Zhang et al. 2020), using IQ-TREE v. 1.6.8 (Nguyen et al. 2015) with 1,000 ultrafast bootstrap replicates (Hoang et al. 2018). The GTR+G4+F model was selected by ModelFinder 2 (Kalyaanamoorthy et al. 2017) according to the corrected Akaike Information Criterion (AICc).

**Table 2.** Samples used in the molecular study.

Species	Voucher number	Accession number	Location
**Ingroups**			
*M.angusta*		MW970280	
		KY349624	
*M.bicruris*	EX_1	PP135569	Mengla, Xishuangbanna, Yunnan
	EX_2	PP135570	
		MW970303	
*M.bisignata*	SY_1	PP135579	Dabie Mountain, Huanggang, Hubei
	SY_3	PP135580	Tangkou, Huangshan, Anhui
	SY_4	PP135581	Jinyun Mountain, Beibei, Chongqing
	SY_5	PP135582	Huangtangxi, Quanzhou, Fujian
	SY_6	PP135583	Yinshan Park, Jinxiu, Guangxi
	SY_7	PP135584	Mangshan Forest Park, Hunan
	SY_8	PP135585	Liupan, Jinhua, Zhejiang
	SY_9	PP135586	Huanglong Mountain, Lushan, Jiangxi
	SY_10	PP135587	Fanjing Mountain, Tongren, Guizhou
	SY_11 (F)	PP135588	E’mei Mountain, Leshan, Sichuan
		MW970310	
		MW970317	
		MW970315	
		KY349596	
		KY349607	
		KY349603	
		KY349604	
*M.bisphaerica* sp. nov.	SP1	PP135563	Shengtang Mountain, Jinxiu, Guangxi
	Q5_34 (F)	PP135562	
*M.caudata*	WB_3	PP135610	Meizi Lake, Pu’er, Yunnan
		MW970283	
		MW970284	
*M.concava*	AY_1	PP135572	Diaoluo Mountain, Lingshui, Hainan
	AY_3 (F)	PP135574	
	AY_4	PP135575	
	AY_2	PP135573	Menglun, Xishuangbanna, Yunnan
	AY_5	PP135576	Maogan, Baoting, Hainan
		KY349650	
		MW970253	
		KY349647	
		MW970254	
		MW970252	
*M.cuspidata*		MW970300	
		MW970301	
*M.deltodonta*	ZT_3	PP135609	Dawei Mountain, Pingbian, Yunnan
		MW970294	
*M.deltodonta*	ZT_3	MW970295	Dawei Mountain, Pingbian, Yunnan
*M.disparilis*		MW970290	
		MW970291	
		MW970292	
*M.forcipata* sp. nov.	HD_3	PP135604	Golden Gully, Zhaoqing, Guangdong
	SHD_1	PP135605	
	SP8 (F)	PP135606	
*M.limbata*		MW970281	
	M2 (F)	PP135607	Dushan, Qiannan, Guizhou
*M.mckittrickae*	MS_3	PP135612	Diaoluo Mountain, Lingshui, Hainan
*M.multipunctata*	DB_1 (F)	PP135566	Menglun, Xishuangbanna, Yunnan
	DB_2 (F)	PP135567	
	DB_3	PP135568	
		KY349646	
		MW970269	
*M.nimbata*		KY349658	
		MW970261	
		MW970259	
		KY349653	
*M.parabisignata* sp. nov.	SY_2	PP135600	Limu Mountain, Qiongzhong, Hainan
	SP4	PP135598	
	SP5 (F)	PP135599	
*M.paratransversa*		MW970262	
		MW970263	
*M.pedata* sp. nov.	NZ_3 (F)	PP135564	Nabang, Yinjiang, Yunnan
	NZ_4 (F)	PP135565	
*M.perspicillaris*	M7	PP135578	Yinggeling, Baisha, Hainan
	H_2	PP135577	
*M.semicircularis* sp. nov.	CY_7	PP135595	Baishaogou, Zunyi, Guizhou
	SP9	PP135596	
	SP10 (N)	PP135597	
*M.speciosa*	HL_3	PP135571	Libo, Qiannan, Guizhou
		KY349620	
		KY349618	
		MW970279	
*M.spinifera*	CY_1	PP135589	Diaoluo Mountain, Lingshui, Hainan
	CY_2	PP135590	
	CY_3	PP135591	Wuyi Mountain, Wuyishan, Fujian
	CY_4	PP135592	Dayao Mountain, Jinxiu, Guangxi
	CY_6	PP135593	Menglun, Xishuangbanna, Yunnan
	M1 (F)	PP135594	Maolan National Forest Park, Guizhou
		KY349628	
		MW970274	
		KY349636	
*M.spinifera*	M1 (F)	KY349639	Maolan National Forest Park, Guizhou
		MW970278	
*M.spinosa*	DC_1 (F)	PP135611	Wuzhi Mountain, Wuzhishan, Hainan
		MW970299	
		KY349617	
		KY349613	
		KY349615	
		KY349610	
*M.transversa*		MW970264	
		MW970265	
		KY349659	
*M.trispinosa*	SC_3 (F)	PP135614	Butterfly Valley, Honghe, Yunnan
	M4	PP135613	
*M.undulata* sp. nov.	SP_2	PP135602	Jinyun Mountain, Beibei, Chongqing
	Q1_29	PP135601	
	SP6 (F)	PP135603	
**Outgroups**			
*Baltavilis*		KT279743	
*Mantisreligiosa*		KM29415	
*Sorineuchoranigra*		MF612149	

Abbreviations: female (F); nymph (N).

We used a molecular species delimitation method (ABGD: Puillandre et al. 2012) to delimit *Margattea* species based on *COI* sequences. ABGD, compared to GMYC and bPTP, provides more conservative estimates, which did not overestimate the number of entities (Puillandre et al. 2012; He et al. 2021). For ABGD, the analysis result was displayed on a web interface (https://bioinfo.mnhn.fr/abi/public/abgd/abgdweb.html). The default parameters were used except for the relative gap width set at 1.0 and using the Jukes-Cantor (JC69) distance.

## ﻿Results

### ﻿Morphological species delimitation based on external morphology

**Figure 1.**
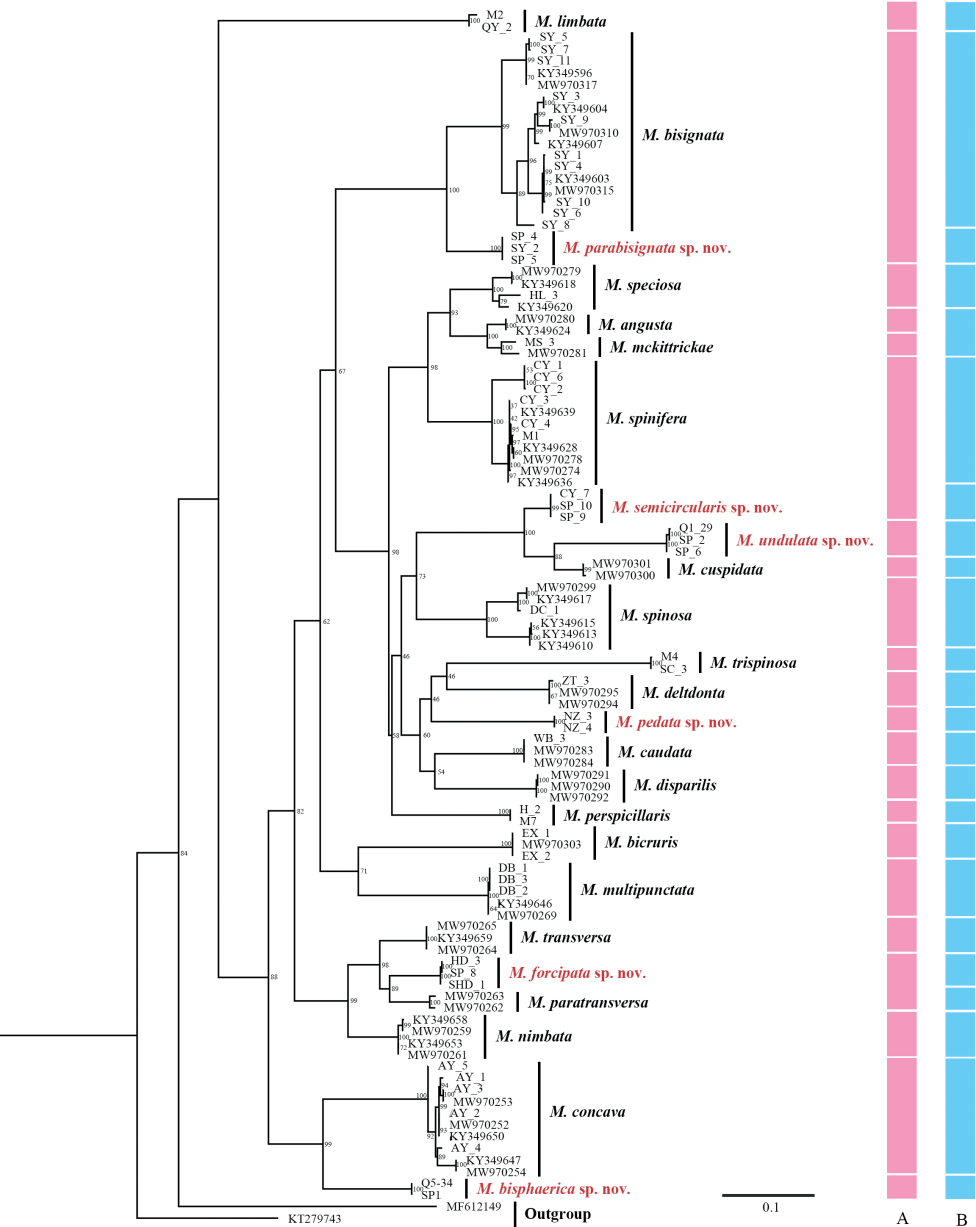
Maximum likelihood (ML) tree and species delimitation of *Margattea* based on *COI* sequence. Branches labels are bootstrap support percentage. Colored bars indicate different species delimitation by different methods **A** morphology (pink) **B** ABGD results (blue).

Combining the external morphological character, we identified 22 morphospecies of *Margattea* from a large number of samples collected, including three new species, *M.pedata* Li & Che, sp. nov., *M.undulata* Li & Che, sp. nov., and *M.bisphaerica* Li & Che, sp. nov. (Fig. 1A).

### ﻿Molecular phylogeny and species delimitation based on COI

In this study, we acquired 105 *COI* sequences of *Margattea* representing 22 morphospecies of *Margattea*. The ML phylogenetic tree showed that samples (including males, females, and nymphs) of the same morphospecies form monophyletic groups, although most of the nodes did not have high bootstrap values (Fig. 1). 24 molecular operational taxonomic units (MOTUs) were delimited by ABGD (Fig. 1B).

### Establishment of three new cryptic species based on molecular data and male genitalia

**Figure 2.**
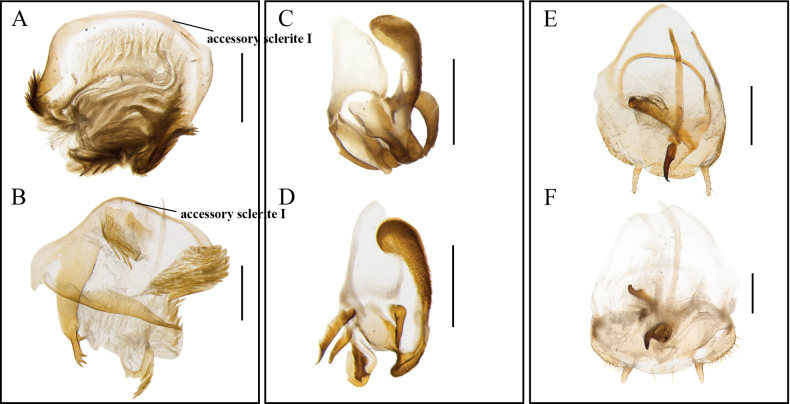
**A***M.spinifera* Bey-Bienko, 1958, median phallomere, dorsal view (CY_2) **B***M.semicircularis* Li & Che, sp. nov., median phallomere, dorsal view (CY_7) **C***M.bisignata* Bey-Bienko, 1970, left phallomere, dorsal view (SY_3) **D***M.parabisignata* Li & Che, sp. nov., left phallomere, dorsal view (SY_2) **E***M.paratransversa* He & Wang, 2021, subgenital plate and median phallomere, dorsal view (MW970262) **F***M.forcipata* Li & Che, sp. nov., subgenital plate and median phallomere, dorsal view (SHD_1). Scale bars: 0.5 mm.

Eighteen of 22 morphological species were well supported by the ABGD result. *M.angusta* Wang, Li, Wang & Che, 2014 and *M.mckittrickae* Wang, Che & Wang, 2009 were considered as one MOTU. He et al. (2021) found some stable morphological differences between the two species, although the genetic distance between them was only ~ 5%. *M.spinifera* Bey-Bienko, 1958, *M.bisignata* Bey-Bienko, 1970, and *M.paratransversa* He & Wang, 2021 were all divided into two MOTUs. These results suggest that it was insufficient and challenging to distinguish the specimens of *Margattea* only based on the external morphological characters. Therefore, we examined the male genitalia of *M.spinifera*, *M.bisignata*, and *M.paratransversa* carefully. For *M.spinifera*, the left end of the accessory sclerite of samples CY_7 and SP9 is trigonate (Fig. 2B), while that of samples CY_1, CY_2, CY_3, CY_4, CY_6, KY349628, MW970274, KY349636, KY349639 and MW970278 is expanded with fuzz (Fig. 2A). For *M.bisignata*, the left phallomere of samples SY_2 and SP4 has a short spiny process (Fig. 2D), while that of samples SY_1, SY_3, SY_4, SY_5, SY_6, SY_7, SY_8, SY_9, SY_10, MW970310, MW970317, MW970315, KY349596, KY349607, KY349603 and KY349604 had a long spine process (Fig. 2C). For *M.paratransversa*, the apex of median phallomere of samples HD_3 and SHD_1 is enlarged and forceps-shaped (Fig. 2F), while that of samples MW970262 and MW970263 has a slightly curved spine (Fig. 2E). In conclusion, three cryptic new species, *M.parabisignata* Li & Che, sp. nov., *M.semicircularis* Li & Che, sp. nov., and *M.forcipata* Li & Che, sp. nov., are discovered mainly based on the male genitalia with the help of the molecular data.

## ﻿Taxonomy

### ﻿ Margattea

Taxon classificationAnimaliaBlattodeaPseudophyllodromiidae

Shelford, 1911

66799326-6CA6-55F6-A508-FE0C083806A0

Margattea Shelford, 1911: 155. Type species: Blattaceylanica Saussure, 1868; by monotypy. Rehn 1931: 302; Bey-Bienko 1938: 121; Bey-Bienko 1950: 145; Princis 1969: 862; Roth 1989: 207; Roth 1991: 980; Wang et al. 2009: 51; Liu and Zhou 2011: 936; Wang et al. 2014: 31; He et al. 2021: 121.
Kuchinga Hebard, 1929: 41. Type species: Phyllodromialongealata Brunner von Wattenwyl, 1898; by selection. Hanitsch 1931: 392. Synonymized by Bey-Bienko 1938: 121. Princis 1969: 862.
Theganosilpha Kumar & Princis, 1978: 33. Type species: Theganopteryxperspicillaris Karny, 1915; by monotypy. Asahina 1979: 119. Synonymized by Roth 1989: 207.
Molestella Bruijning, 1948: 74. Type species: Phyllodromiamolesta Brunner von Wattenwyl & Bruijning, 1948; by monotypy. Princis 1969: 803. Synonymized by Roth 1991: 980.
Margattina Bey-Bienko, 1958: 675. Type species: Margattinatrispina Bey-Bienko, 1958. Synonymized by Liu et Zhou 2011: 936.

#### Diagnosis.

Body small, usually yellowish brown. Interocellar distance slightly wider than the distance between eyes, narrower than the distance between antennal sockets. The fifth maxillary palp expanded, the third and fourth palpi both longer than the fifth palp. Pronotum subelliptical, broader than long, the disc usually with symmetrical maculae and stripes. Tegmina and hind wings fully developed, mostly both extending beyond the end of abdomen. Tegmina with M and CuA radial, M straight with 4–7 branches. Hind wings with ScP and RA expanded at apex, CuA with 4–6 complete branches. Anteroventral margin of front femur Type B_2_ or B_3_. Four proximal tarsomeres with pulvilli. The pretarsi with arolium, tarsal claws symmetrical and specialized, with minute denticles on ventral margins. Eighth abdominal tergum usually specialized, with a tuft of setae in the middle near posterior margin. Supra-anal plate usually short and transverse, paraprocts similar and flaky. Cerci long, with setae on the ventral surface. Male subgenital plate symmetrical or slightly asymmetrical. Styli symmetrical and cylindrical, rarely asymmetrical or non-cylindrical. ***Male genitalia*.** Left phallomere small, irregularly bone-shaped, mostly with spine-like process. Median phallomere slender, rod-shaped, the apex irregular and variable; accessory sclerite complicated, generally arched. Hook phallomere on right side, apex usually curved inwards.

#### Differential diagnosis.

The genus *Margattea* is supposedly closely related to *Chorisoserrata* (Wang et al. 2023) and morphologically similar to *Balta*, but *Margattea* could be distinguished from *Chorisoserrata* and *Balta* by the following characteristics. The genus *Margattea* can be distinguished from *Balta* (Asia, Africa, and parts of Oceania) by the following characteristics: 1) anteroventral margin of front femur Type B_2_ or B_3_, in contrast to C_2_ (but rarely B_3_) in *Balta*; 2) the tarsal claws symmetrical and specialized, but in the latter, the tarsal claws asymmetrical and unspecialized.

The genus *Margattea* can be distinguished from *Chorisoserrata* (parts of Asia and Indonesia) by the following characteristics: 1) anteroventral margin of front femur Type B_2_ or B_3_, in contrast to C_2_ (but rarely B_3_) in *Chorisoserrata*; 2) eighth abdominal tergum usually specialized, with a tuft of setae in the middle near posterior margin; while in the latter, abdominal terga unspecialized.

### Key to species of *Margattea* from China

**Table:** 

1	Tegmina not extending beyond the end of abdomen	**2**
–	Tegmina extending beyond the end of abdomen	**3**
2	Tegmina reaching the middle of abdomen	***M.hemiptera* Bey-Bienko, 1958**
–	Tegmina extending beyond the middle of the abdomen but not reaching the end of abdomen	***M.perspicillaris* (Karny, 1915)**
3	Pronotum without maculae	***M.immaculata* Liu & Zhou, 2011**
–	Pronotum with maculae	**4**
4	The distance between eyes narrow, nearly half of interocellar distance	***M.angusta* Wang, Li, Wang & Che, 2014**
–	The distance between eyes wide, wider than half of interocellar distance	**5**
5	Anteroventral margin of front femur Type B_3_	**6**
–	Anteroventral margin of front femur Type B_2_	**15**
6	Interstylar region nearly truncate, not produced	**7**
–	Interstylar region obviously produced	**9**
7	Styli conical	**8**
–	Styli foot-shaped	***M.pedata* Li & Che, sp. nov.**
8	Median phallomere with three spinelike sclerites	***M.trispinosa* (Bey-Bienko, 1958)**
–	Median phallomere with small spines	***M.mckittrickae* Wang, Che & Wang, 2009**
9	The trailing edge of interstylar region curls upward	***M.furcata* Liu & Zhou, 2011**
–	The trailing edge of interstylar region no curls upward	**10**
10	Interstylar margin semicircular produced	**11**
–	Interstylar margin not semicircular produced	**12**
11	Left phallomere with two small spines	***M.semicircularis* sp. nov.**
–	Left phallomere with three spine-like processes	***M.spinifera* Bey-Bienko, 1958**
12	Two sides of interstylar protrusion curled	**13**
–	Two sides of interstylar protrusion not curled	**14**
13	Interstylar region convex fishtail-shaped	***M.caudata* He & Wang, 2021**
–	Interstylar region convex irregular	***M.disparilis* He & Wang, 2021**
14	Accessory sclerite with a bristle brush at right apex	***M.cuspidata* He & Wang, 2021**
–	Accessory sclerite without a bristle brush at right apex	***M.flexa* Wang, Li, Wang & Che, 2014**
15	Head dark brown or reddish brown	**16**
–	Head yellowish brown	**17**
16	Styli dissimilar	***M.pseudolimbata* Wang, Li, Wang & Che, 2014**
–	Styli similar	***M.limbata* Bey-Bienko, 1954**
17	Pronotal disc with white maculae	***M.multipunctata* Wang, Che & Wang, 2009**
–	Pronotal disc with brown maculae	**18**
18	Interstylar region concave	**19**
–	Interstylar region not concave	**20**
19	Styli symmetrical, conical	***M.concava* Wang, Che & Wang, 2009**
–	Styli asymmetrical, the left shorter than the right	***M.bisphaerica* Li & Che, sp. nov.**
20	Eighth abdominal tergum unspecialized	**21**
–	Eighth abdominal tergum specialized	**22**
21	Posterior margin of supra-anal plate with sharp protrusions	***M.producta* Wang, Che & Wang, 2009**
–	Posterior margin of supra-anal plate without sharp protrusions	***M.punctulata* (Brunner von Wattenwyl, 1893)**
22	Interstylar region with triangular protrusion	***M.deltodonta* He & Wang, 2021**
–	Interstylar region without triangular protrusion	**23**
23	Left phallomere without rodlike structure	**24**
–	Left phallomere with rodlike structure	**25**
24	Apex of median phallomere with sparse brush-like structure composed of similar spines	***M.bisignata* Bey-Bienko, 1970**
–	Apex of median phallomere with sparse brush-like structure composed of uneven spines	***M.parabisignata* Li & Che, sp. nov.**
25	Body overall length not greater than 9.0 mm	**26**
–	Body overall length greater than 12.0 mm	**27**
26	Median phallomere with spinelike sclerite	***M.nimbata* (Shelford, 1907)**
–	Median phallomere without spinelike sclerite	***M.spinosa* Wang, Li, Wang & Che, 2014**
27	Median phallomere with brush structure at apex	**28**
–	Median phallomere without brush structure at apex	29
28	Interstylar margin sinuate	***M.undulata* Li & Che, sp. nov.**
–	Interstylar margin not sinuate	***M.speciosa* Liu & Zhou, 2011**
29	Accessory sclerite of median phallomere with a transverse rod	**30**
–	Accessory sclerite of median phallomere without a transverse rod	***M.bicruris* He & Wang, 2021**
30	Apex of median phallomere enlarged, forceps	***M.forcipata* Li & Che, sp. nov.**
–	Apex of median phallomere with a curved long spine	**31**
31	Left phallomere with three spines	***M.paratransversa* He & Wang, 2021**
–	Left phallomere with four spines	***M.transversa* He & Wang, 2021**

### ﻿ Margattea pedata

Taxon classificationAnimaliaBlattodeaPseudophyllodromiidae

Li & Che sp. nov.

412B5DF4-36C2-57E7-8C53-CBE1CE19A34A

https://zoobank.org/072AC964-F06D-49DC-96F8-35A61A85C467

Fig. 3A–O

#### Type material.

***Holotype***: China • ♂; Yunnan Province, Dehong Dai and Jingpo Autonomous Prefecture, Yingjiang County, Nabang Town; 282 m; 17 Aug. 2015; Xin-Ran Li, Zhi-Wei Qiu leg; SWU-B-PS000001. ***Paratypes***: China • 1 ♂ & 1 ♀; same data as holotype; SWU-B-PS000002–000003 • 5 ♂ & 2 ♀; Yunnan Province, Dehong Dai and Jingpo Autonomous Prefecture, Yingjiang County, Nabang Town; 282 m; 11 Jul. 2012; Dong Wang leg; SWU-B-PS000004–000010.

#### Measurements

**(mm).** Male (*n* = 6), pronotum length × width: 2.4–2.7 × 3.4–3.7, tegmina length: 11.8–12.3, body length: 8.8–10.3, overall length: 13.2–13.7. Female (*n* = 4), pronotum length × width: 2.4–2.9 × 3.2–3.9, tegmina length: 11.2–11.6, body length: 10.9–12.8, overall length: 13.2–14.0.

#### Description.

**Figure 3.**
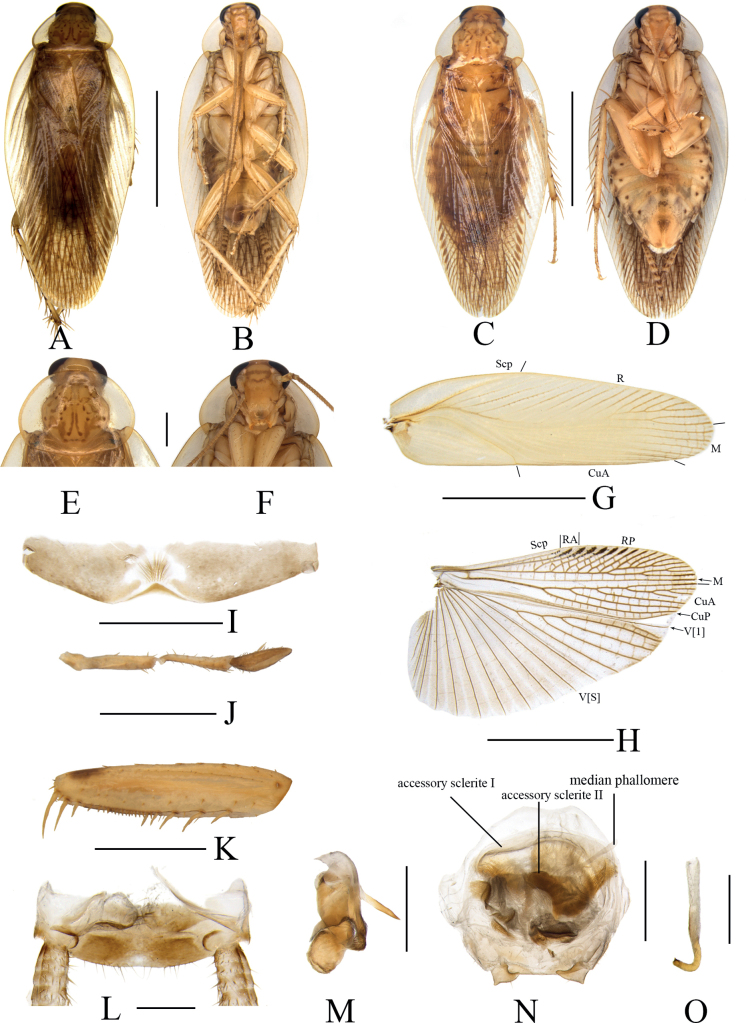
*Margatteapedata* Li & Che, sp. nov. **A, B, E–O** male **C, D** female **A** holotype, dorsal view **B** holotype, ventral view **C** paratype, dorsal view **D** paratype, ventral view **E** pronotum, dorsal view **F** head, ventral view **G** tegmen, ventral view **H** hind wing, ventral view **I** eighth abdominal tergum, ventral view **J** maxillary palpi segments 3–5 **K** front femur, ventral view **L** supra-anal plate, ventral view **M** left phallomere, ventral view **N** subgenital plate and median phallomere, ventral view **O** hook phallomere, ventral view. Scale bars: 5 mm (**A–D, G, H**); 1 mm (**E, F, I–L, N**); 0.5 mm (**M, O**).

**Male. *Coloration*.** Body pale yellow (Fig. 3A, B). Head yellowish brown. Face pale yellow. Interocular space with a wide brown transverse band. Ocellar spots white, interocellar space with a brown band. Antennal base pale yellowish brown, other segments dark brown (Fig. 3E). Maxillary palpi pale brown (Fig. 3J). Pronotal disc pale yellowish brown with reddish tan stripes, and two lateral borders pale linen-colored and transparent (Fig. 3F). Tegmina pale yellowish brown, hind wings brownish grey (Fig. 3G, H). Legs faint yellow. Abdomen yellowish brown, with black stripes along lateral margins of sterna and reaching the end of abdomen; both sides of each abdominal sternum with one small black spot on the inside of the longitudinal lines (Fig. 3B). Cerci yellowish brown to pale brown (Fig. 3L). Styli yellowish white (Fig. 3N).

***Head*.** Vertex slightly exposed, interocellar distance slightly wider than the distance between eyes, narrower than the distance between antennal sockets (Fig. 3F). Fifth maxillary palpus expanded, third and fourth maxillary palpi nearly equal in length, both longer than fifth maxillary palpus (Fig. 3J). Pronotum subelliptical, broader than long, anterior and posterior margins nearly straight, and postero-lateral angle blunt and round; disc with symmetrical spots and stripes (Fig. 3E). ***Tegmina and hind wings*.** Tegmina and hind wings fully developed, both extending beyond the end of abdomen (Fig. 3A, B). Tegmina with M and CuA radial, M straight with three complete branches and one incomplete branch. Hind wings with ScP and RA expanded at apex, M simple, without branches; CuA with four complete branches (Fig. 3G, H). ***Legs*.** Anteroventral margin of front femur Type B_3_ (Fig. 3K). Four proximal tarsomeres with pulvilli. The pretarsi with arolium, tarsal claws symmetrical and specialized, with minute denticles on ventral margins.

***Abdomen and genitalia*.** Eighth abdominal tergum specialized, with a heart-shaped transparent area and a tuft of bristles in the middle (Fig. 3I). Supra-anal plate symmetrical, middle posterior margin slightly concave. Paraprocts simple, similar, and flaky (Fig. 3L). Cerci long, with setae on the ventral surface (Fig. 3L). Styli similar, foot-shaped (Fig. 3N). Subgenital plate nearly symmetrical, posterior margin truncate (Fig. 3N). Left phallomere small, irregular bone-shaped, with a long spine (Fig. 3M). Median phallomere slender rod-shaped, with base curved, apex with a row of spines; accessory sclerite I arched, accessory sclerite II complicated with an inverted bell-shaped structure covered with fuzz (Fig. 3N). Hook phallomere on the right side, apex curved inwards with a short spine (Fig. 3O).

**Female.** Similar to the male (Fig. 3C, D).

#### Diagnosis.

This species is similar to *M.speciosa* Liu & Zhou, 2011 in general appearance, but can be differentiated from the latter by the following characters: 1) styli foot-shaped, while in the latter conical; 2) left phallomere with a long, curved spine, absent in the latter; and 3) accessory sclerite I without a brush-like structure at apex, while in the latter, accessory sclerite I with a brush-like structure at apex.

#### Etymology.

The specific epithet is derived from the Latin word *pedatus*, referring to the foot-shaped styli.

#### Distribution.

China (Yunnan).

### ﻿ Margattea bisphaerica

Taxon classificationAnimaliaBlattodeaPseudophyllodromiidae

Li & Che sp. nov.

F77B1A27-CBAD-5990-A525-C48F07B36D60

https://zoobank.org/753866C7-1196-46B8-BBD0-0C935287AAF2

Fig. 4A–O

#### Type material.

***Holotype***: China • ♂; Guangxi Zhuang Autonomous Region, Laibin City, Jinxiu Yao Autonomous County, Mountain Shengtang; 1182 m; 5 Jun. 2014; Shun-Hua Gui, Xin-Ran Li leg; SWU-B-PS000011. ***Paratypes***: China • 3 ♂ & 1 ♀; same data as holotype; SWU-B-PS000012–000015 • 1 ♂; Guangxi Zhuang Autonomous Region, Laibin City, Jinxiu Yao Autonomous County, Mountain Shengtang; 400 m; 13 Jul. 2015; Lu Qiu, Qi-Kun Bai leg; SWU-B-PS000016.

#### Measurements

**(mm).** Male (*n* = 6), pronotum length × width: 2.3–2.5 × 3.0–3.4, tegmina length: 11.8–12.5, body length: 9.8–11.6, overall length: 13.8–14.9. Female (*n* = 2), pronotum length × width: 2.3–2.5 × 3.3, tegmina length: 10.7–11.4, body length: 10.3–11.0, overall length: 13.8–13.9.

#### Description.

**Figure 4.**
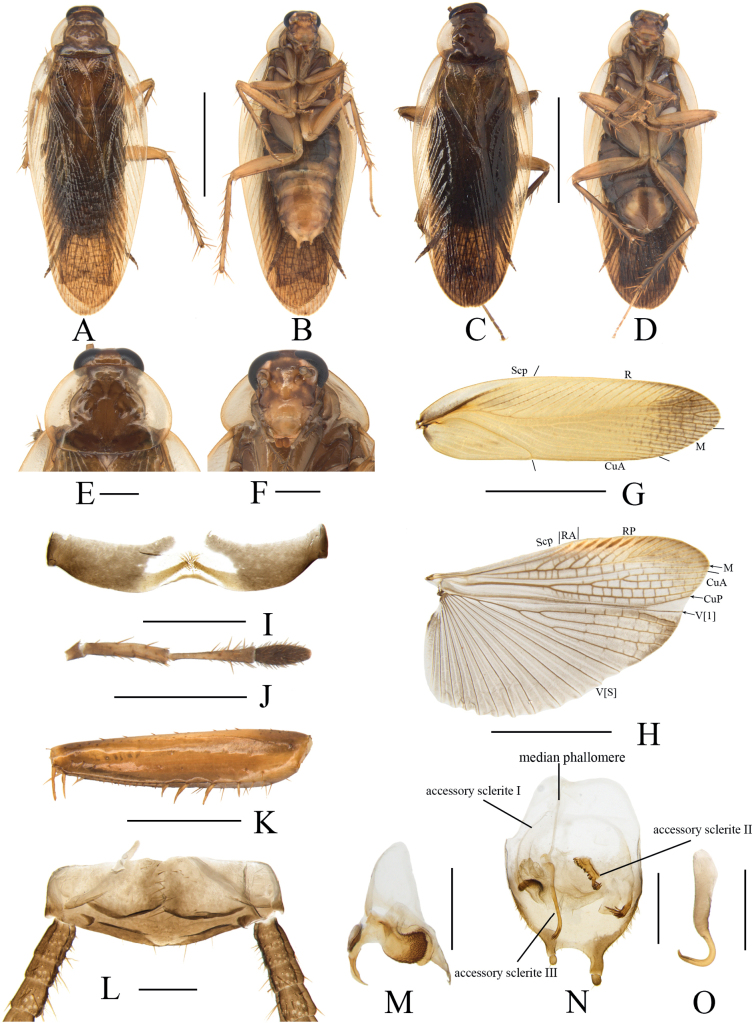
*Margatteabisphaerica* Li & Che, sp. nov. **A, B, E–O** male **C, D** female **A** holotype, dorsal view **B** holotype, ventral view **C** paratype, dorsal view **D** paratype, ventral view **E** pronotum, dorsal view **F** head, ventral view **G** tegmen, ventral view **H** hind wing, ventral view **I** eighth abdominal tergum, ventral view **J** maxillary palpi segments 3–5 **K** front femur, ventral view **L** supra-anal plate, ventral view **M** left phallomere, ventral view **N** subgenital plate and median phallomere, ventral view **O** hook phallomere, ventral view. Scale bars: 5 mm (**A–D, G, H**); 1 mm (**E, F, I–K, N**); 0.5 mm (**L, M, O**).

**Male. *Coloration*.** Body brown (Fig. 4A, B). Head and face yellowish brown. Interocular space with a wide brown transverse band. Ocellar spots yellowish white, interocellar space with a brown band. Antennae blackish brown, antennal space with a brown band (Fig. 4F). Maxillary palpi dark brown (Fig. 4J). Pronotal disc pale brown with dark brown spots and maculae but without stripes, and two lateral borders pale linen-colored and transparent (Fig. 4E). Legs yellowish brown. Tegmina pale yellowish brown, hind wings brownish grey (Fig. 4G, H). Abdomen pale brown. Cerci brown (Fig. 4L). Styli yellowish brown (Fig. 4N).

***Head*.** Vertex slightly exposed, interocellar distance slightly much wider than the distance between eyes, narrower than the distance between antennal sockets (Fig. 4E). Pronotum subelliptical, broader than long, anterior and posterior margins nearly straight, and postero-lateral angle blunt and round; disc with symmetrical but irregular spots and maculae (Fig. 4F). Fifth maxillary palpus expanded, third and fourth maxillary palpi both longer than fifth maxillary palpus (Fig. 4J). ***Tegmina and hind wings*.** Tegmina and hind wings fully developed, both extending beyond the end of abdomen (Fig. 4A, B). Tegmina M and CuA radial, M straight with six complete branches. Hind wings with ScP and RA expanded at apex, M simple, without branches; CuA with four complete branches (Fig. 4 G, H). ***Legs*.** Anteroventral margin of front femur Type B_2_ (Fig. 4K). Four proximal tarsomeres with pulvilli. The pretarsi with arolium, tarsal claws symmetrical and specialized, with minute denticles on ventral margins.

***Abdomen and genitalia*.** Eighth abdominal tergum specialized, with a tuft of bristles in the middle (Fig. 4I). Supra-anal plate symmetrical, anterior margin straight and truncate, the middle of posterior margin slightly concave. Paraprocts simple, similar, and flaky. Cerci long, with setae on the ventral surface (Fig. 4L). Subgenital plate asymmetrical. Styli dissimilar and spherical, the left stylus significantly smaller than the right stylus (Fig. 4N). Left phallomere small, irregular bone-shaped, with a slender curved spine (Fig. 4M). Median phallomere slender rod-shaped, with a curved spine at apex; accessory sclerite I arched, left end expanded, right end with a cluster of thorns; accessory sclerite II brush-shaped; accessory sclerite III slender rod-shaped (Fig. 4N). Hook phallomere on the right side, apex curved hook-shaped (Fig. 4O).

**Female.** Similar to the male. Subgenital plate symmetrical, middle posterior margin concave inward (Fig. 4C, D).

#### Diagnosis.

This species is similar to *M.concava* Wang, Che & Wang, 2009 in general appearance, but can be differentiated from the latter by the following characters: 1) styli dissimilar and spherical, the left stylus significantly smaller than the right stylus; while in the latter, styli similar and conical; 2) left phallomere with a slender curved spine, absent in the latter.

#### Etymology.

The specific name is derived from the Latin words, *bi* and *sphaericus*, referring to the dissimilar and spherical styli.

#### Distribution.

China (Guangxi).

### ﻿ Margattea undulata

Taxon classificationAnimaliaBlattodeaPseudophyllodromiidae

Li & Che sp. nov.

84A9132F-B683-558E-A3E6-76A03CF0D42C

https://zoobank.org/BB55B598-4B51-4F06-ABE0-01005C5A249F

Fig. 5A–O

#### Type material.

***Holotype***: China • ♂; Chongqing City, Beibei District, Mountain Jinyun; 550 m; 12 Jul. 2016; Lu Qiu, Zhi-Wei Qiu leg; SWU-B-PS000017. ***Paratypes***: China • 10 ♂ & 1 ♀; same data as holotype; SWU-B-PS000018–000028 • 2 ♂ & 1 ♀; Chongqing City, Jiangjin District, Mountain Simian; 425 m; 21 Sep. 2007; Wei-Wei Zhang leg; SWU-B-PS000029–000031 • 1 ♂ & 1 ♀; Chongqing City, Liangping District, Dongshan Forest Park; 2 Oct. 2007; Wei-Wei Zhang leg; SWU-B-PS000032–000033.

#### Measurements

**(mm).** Male (*n* = 4), pronotum length × width: 2.4–2.9 × 3.6–3.8, tegmina length: 12.8–13.6, body length: 10.4–12.1, overall length: 14.9–16. Female (*n* = 4), pronotum length × width: 2.3–2.5 × 3.3, tegmina length: 10.7–11.4, body length: 10.3–11.0, overall length: 13.8–13.9.

#### Description.

**Figure 5.**
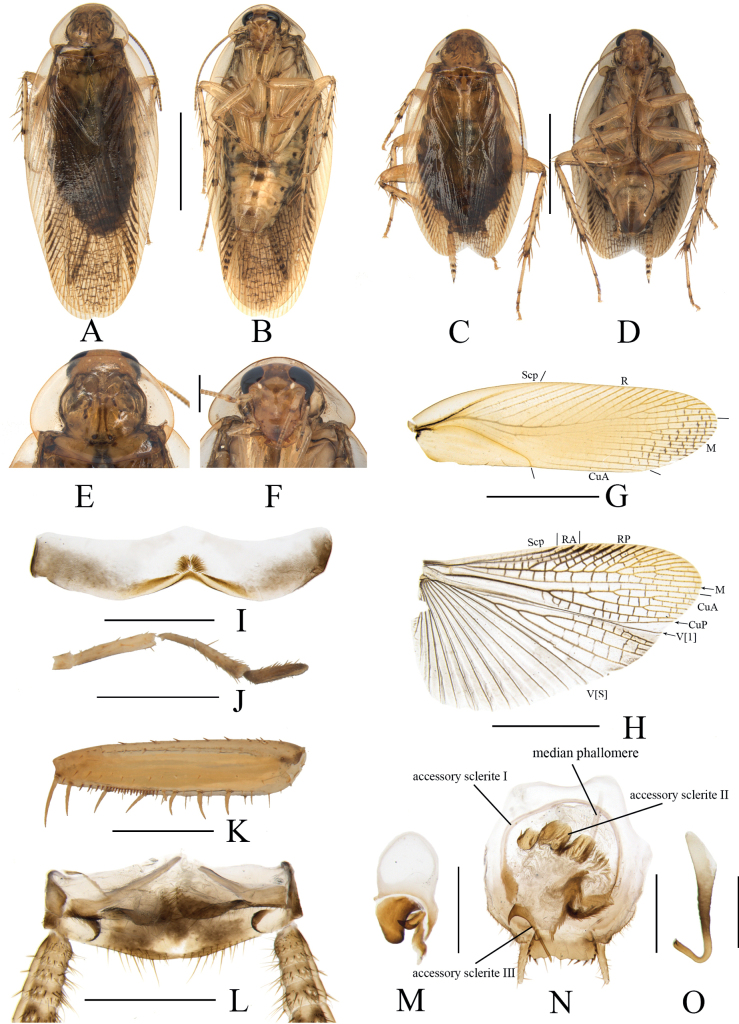
*Margatteaundulata* Li & Che, sp. nov. **A, B, E–O** male **C, D** female **A** holotype, dorsal view **B** holotype, ventral view **C** paratype, dorsal view **D** paratype, ventral view **E** pronotum, dorsal view **F** head, ventral view **G** tegmen **H** hind wing **I** eighth abdominal tergum, ventral view **J** maxillary palpi segments 3–5 **K** front femur, ventral view **L** supra-anal plate, ventral view **M** left phallomere, ventral view **N** subgenital plate and median phallomere, ventral view **O** hook phallomere, ventral view. Scale bars: 5 mm (**A–D, G, H**); 1 mm (**E, F, I–L, N**); 0.5 mm (**M, O**).

**Male. *Coloration*.** Body, head and face yellowish brown (Fig. 5A, B). Interocular space with a brown transverse band. Ocellar spots small, yellowish white. Antennal base yellowish brown, other segments black-brown. The third and fourth maxillary palpi yellowish brown, the fifth palpus maxillary blackish brown (Fig. 5J). Pronotal disc yellowish brown with reddish tan spots and stripes, and two lateral borders pale linen-colored and transparent (Fig. 5E). Legs yellowish brown, with black spots at the base of the tibial spines. Tegmina yellowish brown, hind wings brownish grey (Fig. 5G, H). Abdomen yellowish brown, both sides of each abdominal sternum with one small round black spot on the inside of the longitudinal lines. Cerci yellowish brown (Fig. 5L). Styli pale yellow (Fig. 5N).

***Head*.** Vertex slightly exposed, interocellar distance slightly wider than the distance between eyes, narrower than the distance between antennal sockets (Fig. 5F). Pronotum subelliptical, broader than long, anterior and posterior margins nearly straight, and postero-lateral margin blunt and round; disc with symmetrical spots and stripes (Fig. 5E). Fifth maxillary palpus expanded, third and fourth maxillary palpi nearly equal in length, both twice as long as fifth maxillary palpus (Fig. 5J). ***Tegmina and hind wings*.** Tegmina and hind wings fully developed, both extending beyond the end of abdomen (Fig. 5A, B). Tegmina with M and CuA radial, M straight with seven complete branches. Hind wings with ScP and RA expanded at apex, M simple, without branches; CuA with four complete branches (Fig. 5G, H). ***Legs*.** Anteroventral margin of front femur Type B_3_ (Fig. 5K). Four proximal tarsomeres with pulvilli. The pretarsi with arolium, tarsal claws symmetrical and specialized, with minute denticles on ventral margins. ***Abdomen and genitalia*.** Eighth abdominal tergum specialized, with a tuft of setae near the distinctly concave middle posterior margin (Fig. 5I). Supra-anal plate symmetrical, anterior margin straight and truncate, posterior margin obtusely round. Paraprocts simple, similar and flaky. Cerci long, with setae on the ventral surface (Fig. 5L) Subgenital plate nearly symmetrical, anterior margin distinctly concave in the middle. Styli similar, slender; interstylar margin sinuate, left side with five or six small spines, right side with 5–7 small spines (Fig. 5N). Left phallomere small, irregular bone-shaped, with a small spine (Fig. 5M). Median phallomere slender rod-shaped, with a bristle brush at apex; accessory sclerite I arched, two ends enlarged, right end with a row of spines; accessory sclerite II with three lamellar structures with small spines; accessory sclerite III sickle-shaped (Fig. 5N). Hook phallomere on the right side, apex curved inwards with a short spine (Fig. 5O).

**Female.** Similar to the male but body and wings somewhat shorter (Fig. 5C, D).

#### Diagnosis.

This species is similar to *M.flexa* Wang et al., 2014 in general appearance, but can be differentiated from the latter by the following characters: 1) interstylar margin sinuate, left side with 4–6 small spines, right side with 4–7 small spines; while in the latter, interstylar margin strongly produced, whose lateral sides upturned and scattered with spines; 2) left phallomere irregular bone-shaped, without a small spine; while in the latter, left phallomere irregular bone-shaped, with two spines; 3) accessory sclerite II with three lamellar structures with small spines; while in the latter, accessory sclerite II with lamellar structure without small spines.

#### Etymology.

The specific name is derived from the Latin word *undulatus*, which refers to the sinuate interstylar margin.

#### Distribution.

China (Chongqing).

### ﻿ Margattea semicircularis

Taxon classificationAnimaliaBlattodeaPseudophyllodromiidae

Li & Che sp. nov.

026191B8-D663-5CCF-ADED-8038C0C4BEC0

https://zoobank.org/94D4FBB8-6FD2-43CD-8F41-343E38F269DC

Fig. 6A–N

#### Type material.

***Holotype***: China • ♂; Guizhou Province, Zunyi City, Suiyang County, Qingbantang Town, Baishao Ditch; 30 Jul. 2013; Xiu-Dan Wang leg; SWU-B-PS000034. ***Paratype***: China • 1 ♂; same data as holotype; SWU-B-PS000035.

#### Measurements

**(mm).** Male (*n* = 2), pronotum: length × width 2.3–2.5 × 3.0–3.4, tegmina length: 10.7–11.2, body length: 10.5–11.0, overall length: 12.9–13.2.

#### Description.

**Figure 6.**
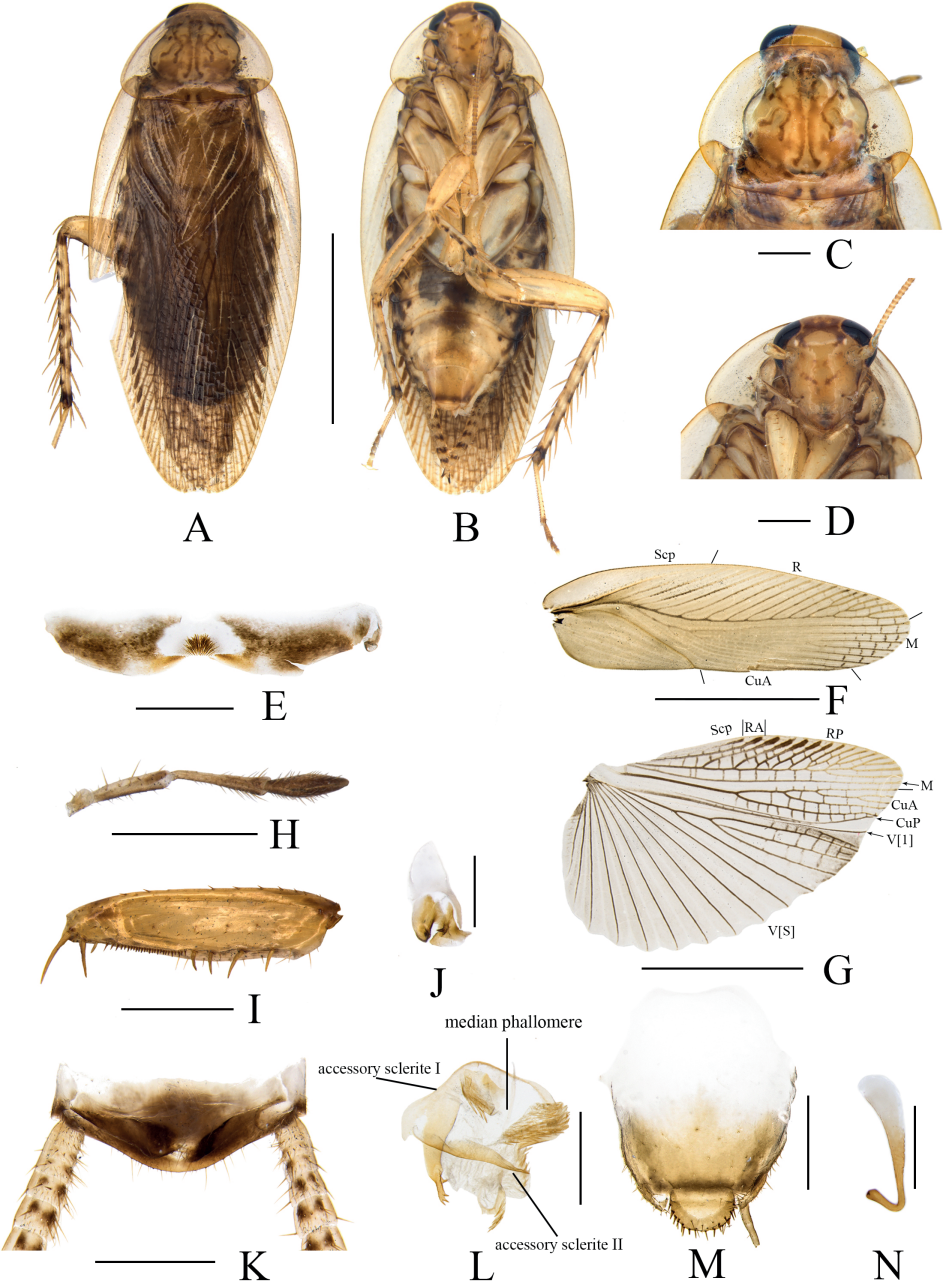
*Margatteasemicircularis* Li & Che, sp. nov. **A–N** male **A** holotype, dorsal view **B** holotype, ventral view **C** pronotum, dorsal view **D** head, ventral view **E** eighth abdominal tergum, ventral view **F** tegmen, ventral view **G** hind wing, ventral view **H** maxillary palpi segments 3–5 **I** front femur, ventral view **J** Left phallomere, dorsal view **K** supra-anal plate, ventral view **L** median phallomere, ventral view **M** subgenital plate, ventral view **N** hook phallomere, ventral view. Scale bars: 5 mm (**A, B, F, G**); 1 mm (**C–E, H, I, K–M**); 0.5 mm (**J, N**).

**Male. *Coloration*.** Body, head and face yellowish brown (Fig. 6A, B). Interocular space with a wide brown transverse band. Ocellar spots small, white, with brown spots beside them. Antennal base pale yellowish brown, other segments brown (Fig. 6D). Maxillary palpi dark brown (Fig. 6H). Pronotal disc yellowish brown with reddish tan spots and stripes, and two lateral borders pale linen-colored and transparent (Fig. 6C). Legs yellowish brown, with black spots at the base of the tibial spines. Tegmina pale yellowish brown, hind wings brownish grey (Fig. 6F, G). Abdomen yellowish brown, with black stripes along lateral margins of sterna and reaching the end of abdomen; both sides of each abdominal sternum with one small black spot on the inside of the longitudinal lines (Fig. 6B). Cerci yellowish brown (Fig. 6K). Styli pale yellow (Fig. 6M).

***Head*.** Vertex slightly exposed, interocellar distance wider than the distance between eyes, narrower than the distance between antennal sockets (Fig. 6C, D). Pronotum subelliptical, broader than long, anterior and posterior margins nearly straight, and postero-lateral angle blunt and round; disc with symmetrical spots and stripes (Fig. 6C). Maxillary palpi slender, fifth maxillary palpus expanded, third and fourth maxillary palpi nearly equal in length, both longer than fifth maxillary palpus (Fig. 6H). ***Tegmina and hind wings*.** Tegmina and hind wings fully developed, both extending beyond the end of abdomen (Fig. 6A, B). Tegmina with M and CuA radial, M straight with seven complete branches. Hind wings with ScP and RA expanded at apex; M simple, without branches; CuA with five complete branches (Fig. 6F, G). ***Legs*.** Anteroventral margin of front femur Type B_3_ (Fig. 6I). Four proximal tarsomeres with pulvilli. The pretarsi with arolium, trsal claws symmetrical and specialized, with minute denticles on ventral margins.

***Abdomen and genitalia*.** Eighth abdominal tergum specialized, with a heart-shaped transparent area and a tuft of bristles in the middle (Fig. 6E). Supra-anal plate symmetrical, anterior margin straight and truncate, the middle of posterior margin slightly produced. Paraprocts simple, similar, and flaky. Cerci long, setae on the ventral surface (Fig. 6K). Subgenital plate nearly symmetrical, anterior margin slightly concave in the middle. Styli similar, conical; interstylar margin strongly semicircular produced, both sides with spines (Fig. 6K). Left phallomere small, irregular, bone-shaped, with two small spines (Fig. 6J). Median phallomere curved hook rod-shaped, with a row of spines at apex. Accessory sclerite I arched, two ends enlarged, left end trigonate, right end with a row of spines; accessory sclerite II long transverse (Fig. 6L, M). Hook phallomere on the right side, apex curved inwards with a short spine (Fig. 6N).

#### Diagnosis.

This species is similar to *M.spinifera* Bey-Bienko, 1958a in general appearance, but can be differentiated from the latter by the following characters: 1) left phallomere with two small spines; while the latter, left phallomere with three spine-like processes; 2) accessory sclerite I arched, left end trigonate; while in the latter, accessory sclerite I arched, left end expanded with fuzz; 3) accessory sclerite II long transverse, and with two lamellar structures with a row of spines, while in the latter, without other accessory sclerites.

#### Etymology.

The scientific name is derived from the Latin word *semicircularis*, which indicates the interstylar margin has a semicircular protrusion.

#### Distribution.

China (Guizhou).

### ﻿ Margattea parabisignata

Taxon classificationAnimaliaBlattodeaPseudophyllodromiidae

Li & Che sp. nov.

1F5DE31E-2E1E-5C57-8494-4AFFAE9A4AA3

https://zoobank.org/7B4FC2A3-9EA9-46F2-AEE8-55B12639511A

Fig. 7A–O

#### Type material.

***Holotype***: China • ♂; Hainan Province, Qiongzhong Li and Miao Autonomous County, Mountain Limu; 600 m; 16 May. 2015; Xin-Ran Li, Zhi-Wei Qiu leg; SWU-B-PS000036. ***Paratypes***: China • 2 ♂ & 1 ♀; same data as holotype; SWU-B-PS000037–000039 • 7 ♂ & 3 ♀; Hainan Province, Qiongzhong Li and Miao Autonomous County, Mountain Limu; 600 m; 16 May. 2015; Xin-Ran Li, Zhi-Wei Qiu leg; SWU-B-PS000040–000049.

#### Measurements

**(mm).** Male (*n* = 7), pronotum length × width: 2.2–2.8 × 3.0–3.6, tegmina length: 11.3–12.2, body length: 10.1–11.1, overall length: 13.0–14.0. Female (*n* = 5), pronotum length × width: 2.2–2.8 × 3.0–3.6, tegmina length: 10.7–11.4, body length: 9.6–11.7, overall length: 12.9–13.7.

#### Description.

**Figure 7.**
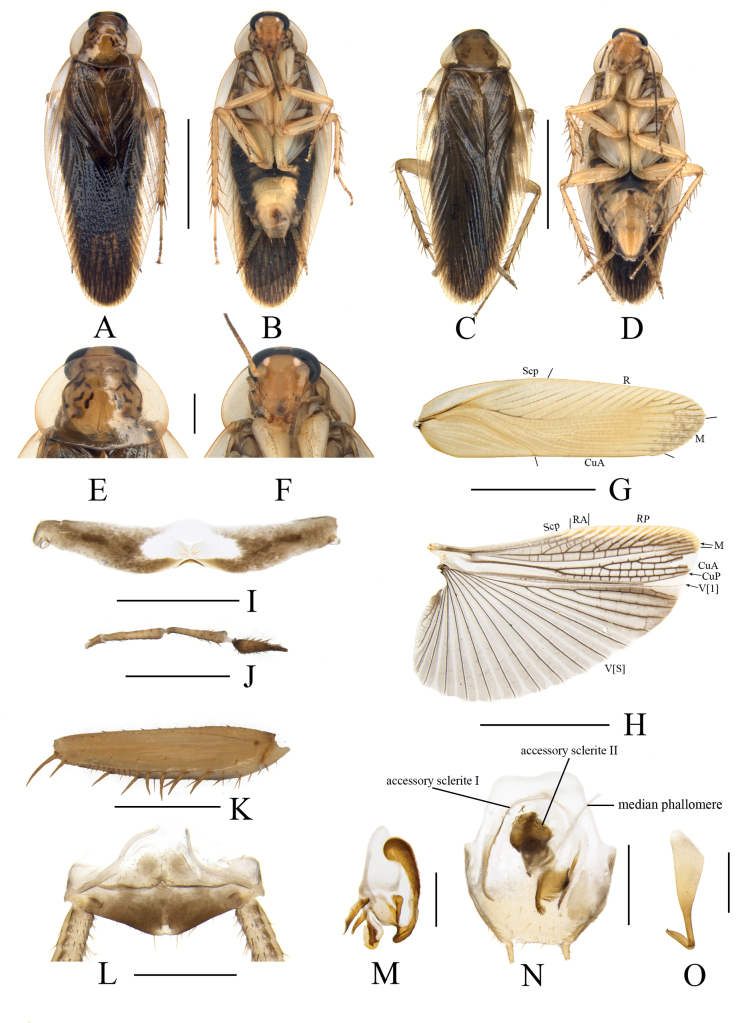
*Margatteaparabisignata* Li & Che, sp. nov. **A, B, E–O** male **C, D** female **A** holotype, dorsal view **B** holotype, ventral view **C** paratype, dorsal view **D** paratype, ventral view **E** pronotum, dorsal view **F** head, ventral view **G** tegmen, ventral view **H** hind wing, ventral view **I** eighth abdominal tergum, ventral view **J** maxillary palpi segments 3–5 **K** front femur, ventral view **L** supra-anal plate, ventral view **M** left phallomere, ventral view **N** subgenital plate and median phallomere, ventral view **O** hook phallomere, ventral view. Scale bars: 5 mm (**A–D, G, H**); 1 mm (**E, F, I–L, N**); 0.5 mm (**M, O**).

**Male. *Coloration*.** Body pale yellowish brown (Fig. 7A, B). Head yellowish brown. Face pale yellow. Interocular space with a brown transverse band. Ocellar spots yellowish white (Fig. 7F). Antennal base pale yellow, other segments yellowish brown. The third and fourth maxillary palpi yellowish brown, the fifth maxillary palpus brown (Fig. 7J). Pronotal disc pale yellowish brown with reddish tan spots but without stripes, and two lateral borders pale linen-colored and transparent (Fig. 7E). Legs faint yellow, with black spots at the base of the tibial spines. Tegmina yellowish brown, hind wings brownish grey (Fig. 7G, H). Abdomen pale yellow, with black stripes along lateral margins of sterna and reaching the end of abdomen. Cerci pale yellow to yellowish brown (Fig. 7L). Styli yellowish brown (Fig. 7N).

***Head*.** Vertex slightly exposed, interocellar distance wider than the distance between eyes, narrower than the distance between antennal sockets (Fig. 7F). Pronotum subelliptical, broader than long, anterior and posterior margins nearly straight, and postero-lateral angle blunt and round; disc with symmetrical spots but without stripes (Fig. 7E). Fifth maxillary palpus expanded, third and fourth maxillary palpi nearly equal in length, both longer than fifth maxillary palpus (Fig. 7J). ***Tegmina and hind wings*.** Tegmina and hind wings fully developed, both extending beyond the end of abdomen (Fig. 7A, B). Tegmina with M and CuA radial, M straight with seven complete branches. Hind wings with ScP and RA expanded at apex, M simple, without branches; CuA with four complete branches (Fig. 7G, H). ***Legs*.** Anteroventral margin of front femur Type B_2_ (Fig. 7K). Four proximal tarsomeres with pulvilli. The pretarsi with arolium, tarsal claws symmetrical and slightly specialized, with minute denticles on ventral margins.

***Abdomen and genitalia*.** Eighth abdominal tergum specialized, with a sparse tuft of bristles in the middle (Fig. 7I). Supra-anal plate symmetrical, the middle of anterior margin slightly concave, posterior margin arcuate produced with setae. Paraprocts simple, similar and flaky, obtuse at apex and each with a spiniform process at the base (Fig. 7L). Subgenital plate nearly symmetrical, anterior margin distinctly concave in the middle, left and right margins both produced in the middle, posterior margin truncate. Styli similar, conical. Cerci long, with setae on the ventral surface (Fig. 7N). Left phallomere large, irregular, bone-shaped, and with spines processes, apex curved upwards with rod-like structure (Fig. 7M). Median phallomere slender rod-shaped, apex with sparse brush-like structure composed of spines of various sizes; accessory sclerite I arched; accessory sclerite II with a lamellar structure with small spines (Fig. 7N). Hook phallomere on the right side, apex curved inwards with a short spine (Fig. 7O).

**Female.** Similar to the male (Fig. 7C, D).

#### Diagnosis.

This species is similar to *M.bisignata* Bey-Bienko, 1970 in general appearance, but can be differentiated from the latter by the following characters: 1) left phallomere with a short spiny process; the latter with a long spine process; 2) median phallomere apex with sparse brush-like structure composed of spines of varying sizes; while in the latter, median phallomere curved at apex, sheet-like, and with brush-shaped structure.

#### Etymology.

The species name *parabisignata* reflects its similarity to *M.bisignata* Bey-Bienko, 1970.

#### Distribution.

China (Hainan).

### ﻿ Margattea forcipata

Taxon classificationAnimaliaBlattodeaPseudophyllodromiidae

Li & Che sp. nov.

8C3216BA-E2DE-574A-8925-A492B4AE05BB

https://zoobank.org/D3F9C77B-A78F-4D6E-A9B1-938A1b5E67EE

Fig. 8A–O

#### Type material.

***Holotype***: China • ♂; Guangdong Province, Zhaoqing City, Fenghuang Town, Jiukeng River, Gold Ditch; 3 Jul. 2015; Zhi-Wei Qiu, Yong-Quan Zhao leg; SWU-B-PS000050. ***Paratypes***: China • 6 ♂ & 1 ♀; same data as holotype; SWU-B-PS000051–000057 • 1 ♂; Guangdong Province, Zhaoqing City, Fenghuang Town, Jiukeng River, Lakeside Villa; 4 Jul. 2015; Zhi-Wei Qiu, Yong-Quan Zhao leg; SWU-B-PS000058.

#### Measurements

**(mm).** Male (*n* = 4), pronotum length × width: 2.4–2.6 × 3.2–3.4, tegmina length: 10.5–11.5, body length: 10.4–10.8, overall length: 13.1–13.4. Female (*n* = 2), pronotum length × width: 2.5–2.7 × 3.4–3.6, tegmina length: 11.1–11.5, body length: 10.6–10.7, overall length: 13.4–13.7.

#### Description.

**Figure 8.**
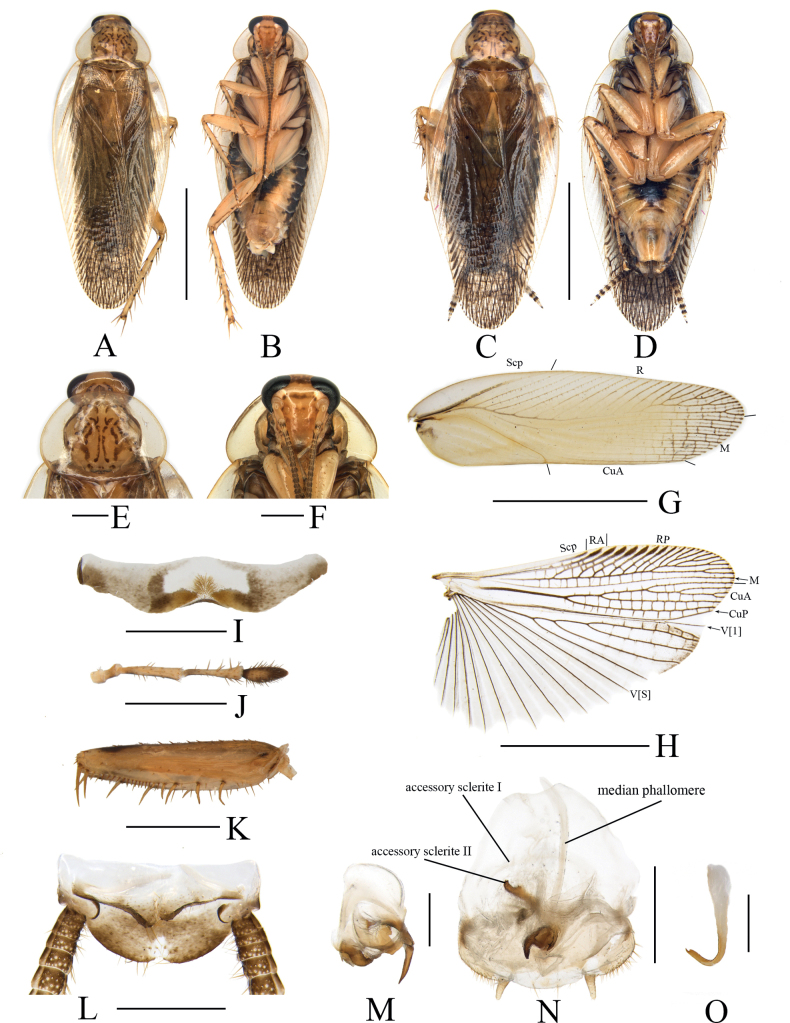
*Margatteaforcipata* Li & Che, sp. nov. **A, B, E–O** male **C, D** female **A** holotype, dorsal view **B** holotype, ventral view **C** paratype, dorsal view **D** paratype, ventral view **E** pronotum, dorsal view **F** head, ventral view **G** tegmen, ventral view **H** hind wing, ventral view **I** eighth abdominal tergum, ventral view **J** maxillary palpi segments 3–5 **K** front femur, ventral view **L** supra-anal plate, ventral view **M** left phallomere, ventral view **N** subgenital plate and median phallomere, ventral view **O** hook phallomere, ventral view. Scale bars: 5 mm (**A–D, G, H**); 1 mm (**E, F, I–L, N**); 0.5 mm (**M, O**).

**Male. *Coloration*.** Body, head and face yellowish brown (Fig. 8A, B). Interocular space with a wider brown transverse band. Ocellar spots big and white (Fig. 8F). Antennal base pale yellow, other segments yellowish brown to brown. Maxillary palpi yellowish brown (Fig. 8J). Pronotal disc yellowish brown with dark brown spots and maculae, and two lateral borders pale linen-colored and transparent (Fig. 8E). Legs yellowish brown, with black spots at the base of the tibial spines. Tegmina pale yellowish brown, hind wings transparent, brownish grey (Fig. 8G, H). Abdomen yellowish brown, with black stripes along lateral margins of sterna and reaching the end of abdomen; both sides of each abdominal sternum with one small round black spot on the inside of the longitudinal lines. Cerci yellowish brown (Fig. 8L). Styli yellowish white (Fig. 8N).

***Head*.** Vertex slightly exposed, interocellar distance wider than the distance between eyes, narrower than the distance between antennal sockets (Fig. 8F). Pronotum subelliptical, broader than long, anterior and posterior margins nearly straight, and postero-lateral angle blunt and round; disc with symmetrical irregular spots and maculae (Fig. 8E). Fifth maxillary palpus expanded, third and fourth maxillary palpi both longer than fifth maxillary palpus (Fig. 8J). ***Tegmina and hind wings*.** Tegmina and hind wings fully developed, both extending beyond the end of abdomen (Fig. 8A, B). Tegmina with M and CuA radial, M straight with six complete branches. Hind wings with ScP and RA expanded at apex, M simple, without branches; CuA with four complete branches (Fig. 8G, H). ***Legs*.** Anteroventral margin of front femur Type B_2_ (Fig. 8K). Four proximal tarsomeres with pulvilli. The pretarsi with arolium, tarsal claws symmetrical and specialized, with minute denticles on ventral margins.

***Abdomen and genitalia*.** Eighth abdominal tergum specialized, with a tuft of bristles in the middle (Fig. 8I). Supra-anal plate symmetrical, anterior margin straight and truncate, posterior margin produced, slightly concave in the middle. Paraprocts simple, similar, and flaky. Cerci long, setae on the ventral surface (Fig. 8L). Subgenital plate nearly symmetrical, anterior margin slightly concave in the middle. Styli similar, slender, conical; interstylar margin irregular produced (Fig. 8N). Left phallomere complex, irregular bone-shaped, with a long spine and three small spines (Fig. 8M). Median phallomere slender rod-shaped, with a forceps-shaped apex. Accessory sclerite I arched; accessory sclerite II with a transverse rod with denticulate (Fig. 8N). Hook phallomere on the right side, apex slightly curved inwards with a short spine (Fig. 8O).

**Female.** Similar to the male.

#### Diagnosis.

This species is similar to *M.transversa* He & Wang, 2021 in general appearance, but can be differentiated from the latter by the following characters: 1) left phallomere with a long spine; the latter with three long spine-like processes; 2) median phallomere with a forceps-shaped apex; while in the latter, median phallomere apex with a curved spine.

#### Etymology.

The specific name *forcipatus*, derived from Latin, refers to the median phallomere with a forceps-shaped apex.

#### Distribution.

China (Guangdong).

## ﻿Discussion

The intraspecific and interspecific genetic distances are considerably high in *Margattea* (Suppl. material 1). The maximum intraspecific genetic distance in this genus (6.6%) existed in two samples of *M.bisignata*, namely SY_1 and SY_7, which showed high similarity in external and genital morphology and were considered conspecific. The interspecific genetic distance (4.8%–33.1%) is much larger than that of other cockroach groups (Blattellidae: *Episymploce*: 6.9%–9.2%; Blattellidae: *Blattella*: 6.7% (Che et al. 2017); Blaberidae: *Cyrtonotula*: 10.6%–13.7% (Wang et al. 2021); Blattidae: *Periplaneta*: 9.9%–13.1% (Luo et al. 2023)). According to recent dating estimates, *Episymploce* and *Periplaneta* diverged from their sister-groups approximately 50 and 40 Ma, respectively, whereas *Margattea* approximately diverged from its sister clade 100 Ma (Liu et al. 2023). We speculate that the large intrageneric genetic distances of *COI* in *Margattea* may be associated with the deep divergence of this genus.

In this study, we initially determined three morphospecies, namely “*M.spinifera*”, “*M.bisignata*”, and “*M.paratransversa*”, whose individuals are almost indistinguishable. In contrast, these morphospecies are each divided into two MOTUs in molecular species delimitation. We hence examined the male genitalia of different samples from each of these morphospecies and found differences in the accessory sclerite I of “*M.spinifera*” (Fig. 2A, B), the left phallomere of “*M.bisignata*” (Fig. 2C, D), and the median phallomere of “*M.paratransversa*” (Fig. 2E, F). With the assistance of male genitalia examination, these MOTUs were determined as different species. This also occurs in other genera in Blattodea, where large genetic distances among closely related species might occur despite small differences in external morphology (Bai et al. 2018; Han et al. 2022; Zhu et al. 2022). Nine *Cryptocercus* species were extremely similar in external morphology, five of which could be distinguished according to chromosome number and female genital characteristics. The other four species could not be distinguished solely based on chromosome number and female genital characteristics, but they could be distinguished by combining these with molecular species definition (Bai et al. 2018). *Anaplectaomei* Bey-Bienko, 1958b could be distinguished from the other three species with very similar external morphology by molecular species definition and female genitalia characteristics (Zhu et al. 2022). *Pseudoeupolyphagasimila* (Qui, 2022) was extremely similar to *Pseudoeupolyphagayunnanensis* (Chopard, 1922) in external morphology, but they could be distinguished by combining female genitalia characteristics, oothecae characteristics, and molecular species definition (Han et al. 2022). In Blattodea and even the insect community, there is an increasing occurrence of closely resembling morphologies that do not necessarily belong to the same species. It is no longer possible to determine species only by morphological characteristics; it is also necessary to recognize species from various aspects, e.g., endosymbionts, cytological characteristics, and ecological characteristics.

## Supplementary Material

XML Treatment for Margattea

XML Treatment for Margattea pedata

XML Treatment for Margattea bisphaerica

XML Treatment for Margattea undulata

XML Treatment for Margattea semicircularis

XML Treatment for Margattea parabisignata

XML Treatment for Margattea forcipata

## ﻿Additional information

### Author ORCIDs

Qian-Qian Li https://orcid.org/0009-0007-3088-9591

Zong-Qing Wang https://orcid.org/0000-0001-9413-1105

Yan-Li Che https://orcid.org/0000-0003-3214-9494

### Data availability

All of the data that support the findings of this study are available in the main text or Supplementary Information.

## Supplementary materials

Supplementary material 1 Interspecific and intraspecific genetic distances Data type xls https://binary.pensoft.net/file/985503This dataset is made available under the Open Database License (http://opendatacommons.org/licenses/odbl/1.0/). The Open Database License (ODbL) is a license agreement intended to allow users to freely share, modify, and use this Dataset while maintaining this same freedom for others, provided that the original source and author(s) are credited.Author: Qian-Qian Li
